# Genistein, a Natural Isoflavone, Alleviates Seizure-Induced Respiratory Arrest in DBA/1 Mice

**DOI:** 10.3389/fneur.2021.761912

**Published:** 2021-11-04

**Authors:** Jialing Guo, Daniel Min, Hua-Jun Feng

**Affiliations:** ^1^Department of Neurology, Xiangya Hospital, Central South University, Changsha, China; ^2^Department of Anesthesia, Critical Care and Pain Medicine, Massachusetts General Hospital, Boston, MA, United States; ^3^Department of Anesthesia, Harvard Medical School, Boston, MA, United States

**Keywords:** SUDEP, S-IRA, dietary supplement, norepinephrine, serotonin, therapeutics

## Abstract

**Objective:** Sudden unexpected death in epilepsy (SUDEP) is a fatal event that ranks second in years of potential life lost among neurological disorders. Seizure-induced respiratory arrest (S-IRA) is the primary instigator leading to death in many SUDEP cases. However, there are currently no effective preventive strategies against S-IRA other than the seizure control. Therefore, it is critical to develop new avenues to prevent SUDEP by investigating the pharmacological interventions of S-IRA. In the present study, we examined the effect of genistein, an isoflavone found in various dietary vegetables, on the incidence of S-IRA in DBA/1 mice.

**Methods:** DBA/1 mice exhibited generalized seizures and S-IRA when subjected to acoustic stimulation. Genistein was intraperitoneally administered alone or in combination with an adrenoceptor antagonist and a serotonin (5-HT) receptor antagonist, respectively. The effects of drug treatments on S-IRA incidence and seizure behaviors were examined.

**Results:** The incidence of S-IRA in DBA/1 mice was significantly reduced 2 h after injection of genistein at 1–90 mg/kg as compared with that in the vehicle control. Genistein could block S-IRA without interfering with any component of seizures, especially at relatively lower dosages. The S-IRA-suppressing effect of genistein was reversed by an α2 adrenoceptor antagonist but was not altered by an α1 antagonist. The inhibitory effect of genistein on S-IRA was not affected by a 5-HT_3_ or 5-HT_2A_ receptor antagonist.

**Significance:** Our data show that genistein reduces S-IRA incidence and can specifically block S-IRA in DBA/1 mice. Its suppressing effect on S-IRA is dependent on activating α2 adrenoceptors. Our study suggests that genistein, a dietary supplement, is potentially useful to prevent SUDEP in at-risk patients.

## Introduction

Sudden unexpected death in epilepsy (SUDEP) ranks second in years of potential life lost among common neurological disorders (1). The lifetime risk of SUDEP is estimated up to 8% in epilepsy patients, and the risk of sudden death is 24- to 28-fold higher in young people with epilepsy than that in the general population (1, 2). Patients with refractory epilepsy are especially vulnerable to SUDEP, with a rate of up to 9 per 1,000 patient-years (3). Multiple pathophysiological mechanisms have been proposed for SUDEP (4–9). However, both clinical and animal studies demonstrated that seizure-induced respiratory arrest (S-IRA) is the major cause of death in SUDEP (10–20). There are currently no effective preventive strategies against S-IRA other than the control of generalized tonic-clonic seizures (21). Therefore, investigating the pathophysiology and pharmacological interventions of S-IRA is of great significance to foster the development of new avenues to prevent SUDEP in patients.

Genistein (4′,5,7-trihydroxyisoflavone) is a natural isoflavone that is found in a variety of dietary vegetables such as soybeans and fava beans (22). As a phytoestrogen, genistein displays a myriad of biological activities and is used for the treatment of many diseases, e.g., post-menopausal syndrome, cancer, cardiovascular disease and diabetes (23–25). Genistein inhibits the reuptake of norepinephrine in human neuroblastoma cells (26); i.e., it is a norepinephrine reuptake inhibitor (NRI). It was reported that genistein enhances serotonin (5-HT) function (27–30) and exerts anticonvulsant effect in rodents (27, 28). Previous studies demonstrated that augmenting the function of certain monoamines (5-HT or norepinephrine) suppresses S-IRA in DBA/1 mice (18, 31–35). Therefore, we hypothesized that genistein could alleviate S-IRA in DBA/1 mice. The aim of this study is to examine the effect of genistein on the incidence of S-IRA and explore the monoaminergic mechanism underlying genistein's action on S-IRA.

## Materials and Methods

### Animals

DBA/1 mice were originally obtained from Envigo (Indianapolis, IN, USA) and were housed and bred in the animal facility under a temperature- and humidity-controlled environment (12-h light/dark cycle) at Massachusetts General Hospital. All animals were provided with rodent food and water *ad libitum*. DBA/1 mice were primed by daily exposure to acoustic stimulation for 3–4 days from post-natal day 26. Once a DBA/1 mouse displayed S-IRA after generalized audiogenic seizures and was resuscitated (see below), it became susceptible to S-IRA in subsequent tests. Primed DBA/1 mice of both sexes at ~2 months of age were used in the experiments. The animal protocol (# 2012N000024) was approved by the Massachusetts General Hospital Institutional Animal Care and Use Committee. Every effort was made to reduce the stress of the animals and minimize the number of the animals used in experiments.

### Drugs

Genistein (G6649), prazosin hydrochloride (P7791), yohimbine hydrochloride (Y3125), and ondansetron hydrochloride dihydrate (O3639) were purchased from Millipore Sigma (St. Louis, MO, USA), and ketanserin tartrate (0908) was obtained from R&D Systems (Minneapolis, MN, USA). Genistein was dissolved in 60% dimethyl sulfoxide (DMSO) and 40% saline (0.9% NaCl), and all other agents were dissolved in saline.

### Effects on S-IRA of Genistein and Genistein in Combination With an Adrenoceptor Antagonist or a 5-HT Receptor Antagonist

As previously described (36), a primed DBA/1 mouse was placed in a cylindrical plexiglass chamber, and audiogenic seizures were evoked by acoustic stimulation using an electric bell (96 dB SPL) in a sound-isolated room. The acoustic stimulus was presented for up to 1 min or until the mouse exhibited wild running and generalized tonic-clonic seizures followed by S-IRA. Once S-IRA occurred, the mouse was resuscitated using a rodent respirator (Harvard Apparatus 680, Holliston, MA, USA) (7).

S-IRA susceptibility was confirmed in all primed DBA/1 mice 24 h prior to the experiment. The effect of genistein or vehicle on the incidence of S-IRA and seizure behaviors was tested by intraperitoneal (i.p.) injection of various dosages of genistein or the vehicle (10 ml/kg) 2 h prior to acoustic stimulation. To assess whether the noradrenergic or 5-HT signaling was involved in the action of genistein, an adrenoceptor antagonist or a 5-HT receptor antagonist was administered 30 min prior to genistein injection, and acoustic stimulation was applied 2 h after genistein injection. The effect of the antagonist alone on S-IRA was examined by i.p. administration of the drug 2.5 h prior to acoustic stimulation.

Audiogenic seizures and S-IRA were digitally recorded using a video camera for offline analysis. For those mice in which S-IRA was blocked during the test, recovery of the susceptibility to S-IRA was examined at 24-h intervals after the test until it returned.

### Statistical Analysis

Statistical analysis was performed using Prism 5.0d software (GraphPad Software Inc., La Jolla, CA, USA). S-IRA incidence between the treatment and vehicle group or between the treatment groups was compared using Chi-square test (two-tailed). Statistical significance was considered if *p* < 0.05.

## Results

### Genistein Reduces S-IRA in DBA/1 Mice

**Figure 1:**
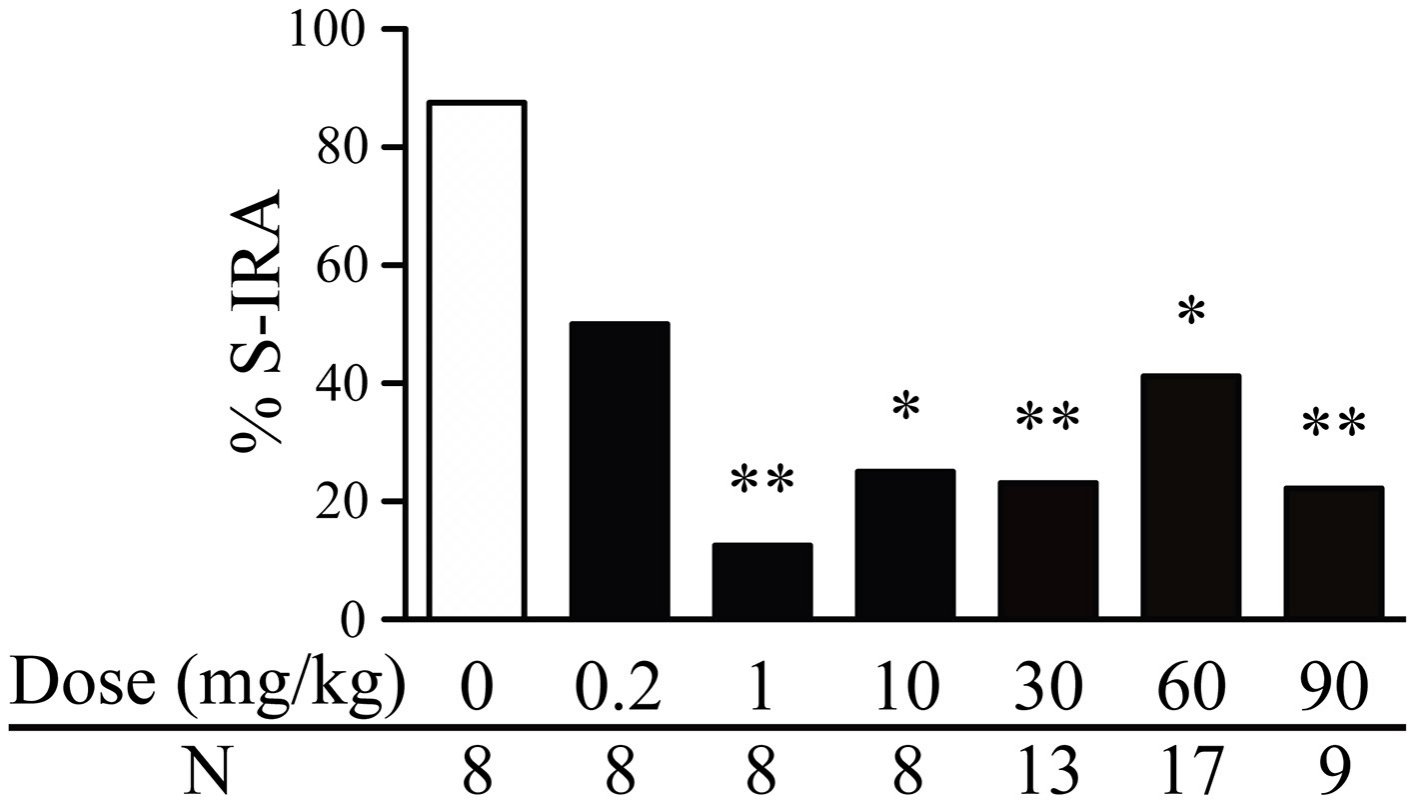
Genistein reduces the incidence of S-IRA in DBA/1 mice. Compared with vehicle control (dose zero), systemic administration of genistein at 1–90 mg/kg significantly decreased the incidence of S-IRA in DBA/1 mice. S-IRA incidence was not significantly altered by genistein at 0.2 mg/kg compared with vehicle control. *p < 0.05; **p < 0.01: Significantly different from the vehicle control (dose zero).

The incidence of S-IRA in DBA/1 mice was significantly reduced 2 h after i.p. administration of genistein at 1 mg/kg (12.5%, *n* = 8; *p* < 0.01), 10 mg/kg (25%, *n* = 8; *p* < 0.05), 30 mg/kg (23.1%, *n* = 13; *p* < 0.01), 60 mg/kg (41.2%, *n* = 17; *p* < 0.05), and 90 mg/kg (22.2%, *n* = 9; *p* < 0.01) as compared with that in the vehicle control (87.5%, *n* = 8). However, compared with that in the vehicle control, the incidence of S-IRA was not significantly suppressed by genistein at 0.2 mg/kg (50%, *n* = 8) (Figure 1).

Among those DBA/1 mice whose S-IRA was blocked by genistein, genistein at 1 mg/kg inhibited S-IRA without interfering with any component of audiogenic seizures in 100% of mice, suggesting that genistein specifically blocks S-IRA in these mice. Genistein specifically blocked S-IRA in 100% of mice at 10 mg/kg, 90% at 30 mg/kg, 80% at 60 mg/kg and 42.9% at 90 mg/kg.

At relatively higher dosages, genistein also produced a considerable anticonvulsant effect. Genistein blocked tonic-clonic seizures in 7.7% of mice at 30 mg/kg, 11.8% of mice at 60 mg/kg and 44.4% of mice at 90 mg/kg. At these dosages of genistein, wild running seizures were still observed in the majority of mice; 92.3% at 30 mg/kg, 88.2% at 60 mg/kg and 88.9% at 90 mg/kg.

### The S-IRA-Suppressing Effect of Genistein Is Reversed by an α2 Adrenoceptor Antagonist

**Figure 2:**
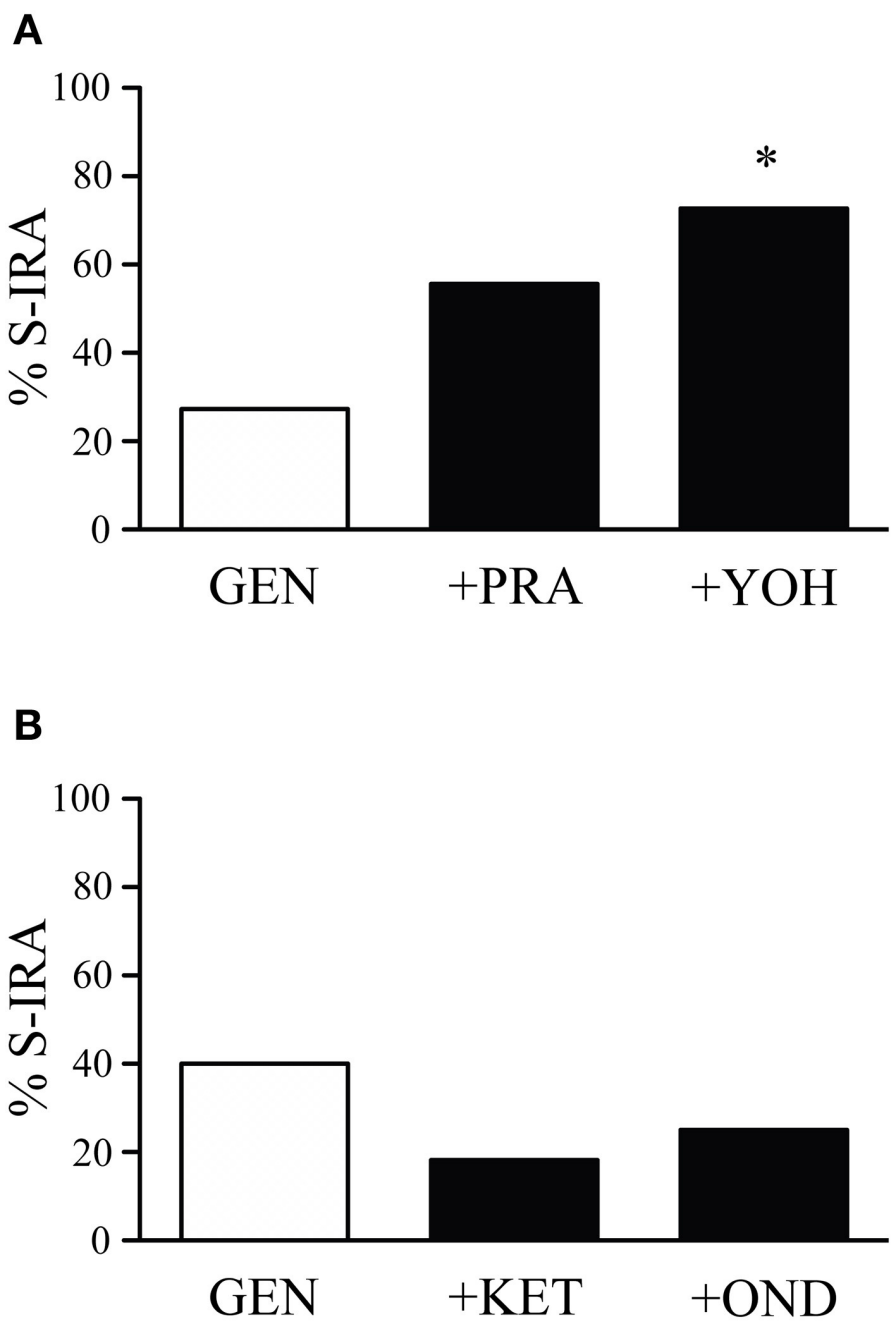
The suppressing effect of genistein on S-IRA in DBA/1 mice is prevented by an α2 adrenoceptor antagonist but not by an α1 adrenoceptor antagonist as well as a 5-HT_2A_ or 5-HT_3_ receptor antagonist. **(A)** Compared with the incidence of S-IRA evoked by genistein alone (1 mg/kg), administration of the α2 adrenoceptor antagonist yohimbine (5 mg/kg) 30 min prior to genistein injection (1 mg/kg) significantly elevated S-IRA incidence. Administration of the α1 adrenoceptor antagonist prazosin (1 mg/kg) 30 min before genistein injection (1 mg/kg) did not significantly change S-IRA incidence compared with that evoked by genistein alone (1 mg/kg). **(B)** Compared with the incidence of S-IRA evoked by genistein alone (1 mg/kg), administration of the 5-HT_2A_ receptor antagonist ketanserin (0.5 mg/kg) or the 5-HT_3_ receptor antagonist ondansetron (2 mg/kg) 30 min prior to genistein injection (1 mg/kg) did not significantly alter S-IRA incidence. *p < 0.05: Significantly different from the effect on S-IRA evoked by genistein alone.

The incidence of S-IRA tested 2.5 h after administration of the α1 adrenoceptor antagonist prazosin alone (1 mg/kg) (87.5%, *n* = 8) or the α2 adrenoceptor antagonist yohimbine alone (5 mg/kg) (100%, *n* = 8) was not significantly different from tests 24 h prior to the experiment (100%). Compared with the incidence of S-IRA in the presence of 1 mg/kg genistein alone (27.3%, *n* = 22), administration of yohimbine (5 mg/kg) 30 min prior to genistein injection (1 mg/kg) significantly increased S-IRA incidence to 72.7% (*n* = 11; *p* < 0.05). Although administration of prazosin (1 mg/kg) 30 min before genistein injection (1 mg/kg) also enhanced S-IRA incidence, the enhancement was not significantly different from that evoked by genistein alone (1 mg/kg) (*p* = 0.135) (Figure 2A).

Compared with the incidence of S-IRA tested 24 h prior to the experiment (100%), administration of the 5-HT_2A_ receptor antagonist ketanserin alone (0.5 mg/kg) (75%, *n* = 8) or the 5-HT_3_ receptor antagonist ondansetron alone (2 mg/kg) (87.5%, *n* = 8) 2.5 h prior to acoustic stimulation did not significantly alter S-IRA incidence. Administration of ketanserin (0.5 mg/kg) or ondansetron (2 mg/kg) 30 min ahead of genistein injection (1 mg/kg) did not significantly change S-IRA incidence as compared with that evoked by genistein alone (1 mg/kg) (Figure 2B).

## Discussion

We report in this study that systemic administration of genistein, a compound found in many dietary vegetables and a dietary supplement widely available over the counter, lowers the incidence of S-IRA and specifically blocks S-IRA in DBA/1 mice. The S-IRA-suppressing effect of genistein is reversed by an α2 adrenoceptor antagonist but not by an α1 antagonist or 5-HT receptor antagonists, suggesting that genistein reduces S-IRA incidence by stimulating α2 adrenoceptors.

### Genistein as a Dietary Supplement Reduces S-IRA Incidence and Specifically Blocks S-IRA in DBA/1 Mice

It is urgent to develop effective strategies to prevent SUDEP (1). One useful approach to impeding SUDEP is the pharmacological treatment to minimize the risk of generalized tonic-clonic seizures, a seizure type that triggers the majority of witnessed SUDEP (37, 38). Although various antiseizure drugs are available for control of seizures, approximately one-third of patients are refractory to the treatment with current medications (39). Thus, it is very important to develop new avenues for treatment of epilepsy and SUDEP, as the SUDEP rate is high for patients with refractory epilepsy (3).

It was reported that 5-hydroxytryptophan (5-HTP), a precursor for 5-HT synthesis, suppresses S-IRA in DBA/1 mice (18). 5-HTP is extracted from the seeds of a plant known as *Griffonia simplicifolia* and is widely used as an over-the-counter dietary supplement to relieve the symptoms of 5-HT-related disorders (40). In the current study, we observed that genistein reduces the incidence of S-IRA in DBA/1 mice. Also as an over-the-counter dietary supplement, genistein is advantageous to 5-HTP in its easy availability. Genistein is present in the fruit of a plant named *Sophora japonica* Leguminosae (41). Interestingly, it is abundantly found in a variety of dietary vegetables, including soybeans, lupin and fava beans, which are widely consumed by people around the world (42, 43).

It appears that genistein effect on S-IRA is not clearly dose-dependent. The reasons underlying this lack of dose-response for genistein action are unknown. However, it was reported that selective serotonin reuptake inhibitors (SSRIs) are not dose-dependent when used as antidepressants (44, 45), which may be due to complicated factors that include autoreceptor regulation (46, 47). As genistein is an NRI, similar factors may contribute to the current observation that genistein reduces S-IRA in a relatively dose-independent manner.

Genistein can selectively block S-IRA without interrupting any component of audiogenic seizures, especially at relatively lower dosages. This finding is reminiscent of the effects of 5-HT-enhancing agents such as SSRIs and NRIs on S-IRA. It was reported that several SSRIs including fluoxetine and sertraline (31, 36, 48, 49) and 5-HTP (18) can specifically inhibit S-IRA. The NRI atomoxetine can also selectively block S-IRA (33, 34). The selective inhibition of S-IRA by genistein indicates that it may achieve this effect via stimulating noradrenergic and/or 5-HT neurotransmission.

### Noradrenergic but Not 5-HT Neurotransmission Is Involved in the Inhibition of S-IRA by Genistein

How does genistein work to suppress S-IRA in DBA/1 mice? Our study demonstrated that the inhibitory effect of genistein on S-IRA was prevented by the α2 adrenoceptor antagonist yohimbine but was not significantly altered by the α1 adrenoceptor antagonist prazosin. These findings indicate that genistein reduces S-IRA by enhancing noradrenergic neurotransmission and activating α2 adrenoceptors. Consistent with this, it was reported that genistein inhibits the reuptake of norepinephrine by reducing the activity of norepinephrine transporters in human neuroblastoma cells (26), indicating that genistein is an NRI. Interestingly, our recent data show that the traditional NRI atomoxetine reduces S-IRA via activation of α2 adrenoceptors (50). α2 adrenoceptors are found both pre-synaptically and post-synaptically in the CNS (51). Genistein alleviates S-IRA in DBA/1 mice likely through the activation of post-synaptic α2 adrenoceptors, as pre-synaptic α2 adrenoceptors are autoreceptors, and activation of these receptors would inhibit norepinephrine release (51).

In the present study, we observed that the S-IRA-suppressing effect of genistein cannot be reversed by antagonists of 5-HT_2A_ and 5-HT_3_ receptors, which were previously shown to be involved in S-IRA in provoked seizure models (17, 32, 35). This observation suggests it is unlikely that 5-HT neurotransmission contributes to genistein inhibition of S-IRA in DBA/1 mice. Genistein was reported to augment 5-HT neurotransmission (27–30) and engender anticonvulsant effects in rodents through the 5-HT system (27, 28). In line with this, we found that genistein at relatively high dosages produces anticonvulsant effects in DBA/1 mice. Further studies are needed to explore if 5-HT signaling is involved in the anticonvulsant action of genistein in this animal model.

In the current study, we only focused on the pharmacologic effect of genistein on S-IRA by systemic administration of this compound. More studies are needed in the future to investigate the detailed action of genistein such as the molecular mechanism underlying genistein regulation of S-IRA and the nuclei where genistein exerts its effect in the brain.

## Conclusions

In summary, our data demonstrate that genistein, a natural diet supplement, reduces the incidence of S-IRA in DBA/1 mice. It can selectively block S-IRA without interrupting any component of seizures at relatively lower dosages and produces anticonvulsant effects at relatively higher dosages. The suppressing effect of genistein on S-IRA is dependent on the activation of α2 adrenoceptors. These data suggest that genistein is potentially useful to prevent SUDEP in at-risk patients.

## Data Availability Statement

The raw data supporting the conclusions of this article will be made available by the authors, without undue reservation.

## Ethics Statement

The animal study was reviewed and approved by Massachusetts General Hospital Institutional Animal Care and Use Committee (IACUC).

## Author Contributions

JG carried out experiments, performed data analysis, and drafted the manuscript. DM performed experiments. H-JF conceptualized the idea and wrote and edited the manuscript. All authors contributed to the article and approved the submitted version.

## Funding

This work was supported by NIH R01 NS112319 and the fund from the Massachusetts General Hospital Department of Anesthesia, Critical Care and Pain Medicine to H-JF. JG was a recipient of the graduate fellowship from China Scholarship Council.

## Conflict of Interest

The authors declare that the research was conducted in the absence of any commercial or financial relationships that could be construed as a potential conflict of interest.

## Publisher's Note

All claims expressed in this article are solely those of the authors and do not necessarily represent those of their affiliated organizations, or those of the publisher, the editors and the reviewers. Any product that may be evaluated in this article, or claim that may be made by its manufacturer, is not guaranteed or endorsed by the publisher.

## Abbreviations

5-HT: Serotonin
5-HTP: 5-Hydroxytryptophan
i.p.: Intraperitoneal(ly)
NRI: Norepinephrine reuptake inhibitor
S-IRA: Seizure-induced respiratory arrest
SSRI: Selective serotonin reuptake inhibitor
SUDEP: Sudden unexpected death in epilepsy.
